# H3K27 Demethylase, JMJD3, Regulates Fragmentation of Spermatogonial Cysts

**DOI:** 10.1371/journal.pone.0072689

**Published:** 2013-08-15

**Authors:** Naoki Iwamori, Tokuko Iwamori, Martin M. Matzuk

**Affiliations:** 1 Departments of Pathology & Immunology, Baylor College of Medicine, Houston, Texas, United States of America; 2 Department of Molecular and Cellular Biology, Baylor College of Medicine, Houston, Texas, United States of America; 3 Department of Molecular and Human Genetics, Baylor College of Medicine, Houston, Texas, United States of America; 4 Department of Pharmacology, Baylor College of Medicine, Houston, Texas, United States of America; 5 Center for Reproductive Medicine, Baylor College of Medicine, Houston, Texas, United States of America; 6 Center for Drug Discovery, Baylor College of Medicine, Houston, Texas, United States of America; 7 Center of Biomedical Research, Research Center for Human Disease Modeling, Graduate School of Medical Sciences, Kyushu University, Fukuoka-shi, Fukuoka, Japan; 8 Laboratory of Biomedicine, Division of Pathobiology, Department of Basic Medicine, Faculty of Medicine, Kyushu University, Fukuoka-shi, Fukuoka, Japan; 9 Division of Organogenesis and Regeneration, Medical Institute of Bioregulation, Kyushu University, Fukuoka-shi, Fukuoka, Japan; University Hospital of Münster, Germany

## Abstract

The spermatogonial stem cell (SSC) compartment is maintained by self-renewal of stem cells as well as fragmentation of differentiating spermatogonia through abscission of intercellular bridges in a random and stochastic manner. The molecular mechanisms that regulate this reversible developmental lineage remain to be elucidated. Here, we show that histone H3K27 demethylase, JMJD3 (KDM6B), regulates the fragmentation of spermatogonial cysts. Down-regulation of *Jmjd3* in SSCs promotes an increase in undifferentiated spermatogonia but does not affect their differentiation. Germ cell-specific *Jmjd3* null male mice have larger testes and sire offspring for a longer period compared to controls, likely secondary to increased and prolonged maintenance of the spermatogonial compartment. Moreover, JMJD3 deficiency induces frequent fragmentation of spermatogonial cysts by abscission of intercellular bridges. These results suggest that JMJD3 controls the spermatogonial compartment through the regulation of fragmentation of spermatogonial cysts and this mechanism may be involved in maintenance of diverse stem cell niches.

## Introduction

Spermatogenesis occurs throughout most of the adult lifetime of most males and is supported by a small subset of undifferentiated spermatogonial stem cells (SSCs) that can self-renew and also produce differentiated progeny continuously [1,2]. During spermatogenesis, all germ cells are connected with neighboring sister germ cells by a unique structure called the intercellular bridge. Visualization of intercellular bridges allows one to distinguish the early stages of postnatal spermatogenesis as spermatogonia initiate the process of differentiation [3]. Most primitive spermatogonia exist as A_single_ (A_s_, single spermatogonia). As germ cells begin to differentiate, they form A_paired_ (A_pr_, two spermatogonia connected by an intercellular bridge) and A_aligned_ (A_al_, 4, 8, or 16 spermatogonia connected by intercellular bridges). Spermatogonia connected in chains longer than 16 are believed to be committed to differentiation [4,5]. In the primate, there are two types of spermatogonia, A_dark_ and A_pale_ spermatogonia. A_dark_ spermatogonia function as reserve stem cells that rarely divide and replenish progenitor cell compartment in case of injury or disease, whereas A_pale_ spermatogonia are progenitors, in which germ cells expand their numbers by mitotic proliferation [6,7]. The relationship between the length of spermatogonial chains and differentiation status has not been clarified yet.

To date, a number of intrinsic as well as extrinsic factors, including cell surface markers, transcription factors, and other proteins, have been identified as essential proteins that regulate self-renewal or differentiation of SSCs [1]. However, epigenetic mechanisms regulating SSCs have not been well characterized. Among several epigenetic modifications, which include DNA methylation and histone modifications, methylation of lysine 27 of histone H3 (H3K27) is implicated in embryonic development as well as differentiation of stem cells. H3K27 is tri-methylated by Enhancer of Zest Homologue 2 (EZH2, also called KMT6), a catalytic component of polycomb repressive complex 2 (PRC2), and is associated with repression of gene transcription. Most targets of PRC2 are genes essential for development in embryonic stem cells, stem cell maintenance, and pluripotency of differentiated cells [8,9]. Loss of any core components of PRC2 subunits (EZH2, EED, or SUZ12) results in a developmental block at the gastrula stage [10]. Although PRC2 components suppress differentiation of stem cells, they are not required for stem cell maintenance [11,12]. H3K27 methylation is removed by UTX and JMJD3 (also called KDM6A and KDM6B, respectively). UTX is ubiquitously expressed and escapes X-inactivation and regulates HOX gene activation and posterior development [13,14]. Moreover, recent studies show that UTX is involved in myogenesis, cardiac development and epigenetic reprogramming [15,16]. Alternatively, JMJD3 is predominantly expressed in stem cells and regulates differentiation and dedifferentiation in neural and epidermal differentiation, skin repair, and inflammation [17-20]. In the testis, the expression and role of JMJD3 remains to be elucidated, although dramatic changes of H3K27 methylation during spermatogenesis have been demonstrated [21].

It was believed that SSCs were homogeneous and that differentiating spermatogonia, which are connected with neighboring cells after cell division through intercellular bridges, was not reversible, although it is well known in the *Drosophila* germline stem cell system that differentiating germ cells can dedifferentiate into stem cells by abscission of intercellular bridges [22,23]. However, three research groups demonstrated that differentiating mouse spermatogonia could revert into undifferentiated spermatogonia by abscission of intercellular bridges and that the SSC compartment is rapidly and stochastically replaced by conversion of differentiating spermatogonia into undifferentiated spermatogonia [24-26].

In this report, we first examined localization of JMJD3 in the testis and found that JMJD3 was preferentially localized in PLZF-positive undifferentiated spermatogonia. Next, we generated a mouse model in which *Jmjd3* was specifically depleted in germ cells to examine if JMJD3 is required for self-renewal or differentiation of SSCs. Although germ cell-specific *Jmjd3* null male mice are fertile, they sire pups for a longer period than controls. We also found that some spermatogonia are separated from the growing colony by disruption of intercellular bridges. Compartments of undifferentiated spermatogonia were increased by JMJD3 deletion, likely secondary to fragmentation of differentiating spermatogonia. Our data suggest that JMJD3 controls spermatogonial compartment by the regulation of fragmentation of spermatogonial cysts.

## Results

### Preferential expression of JMJD3 in undifferentiated spermatogonia

**Figure 1:**
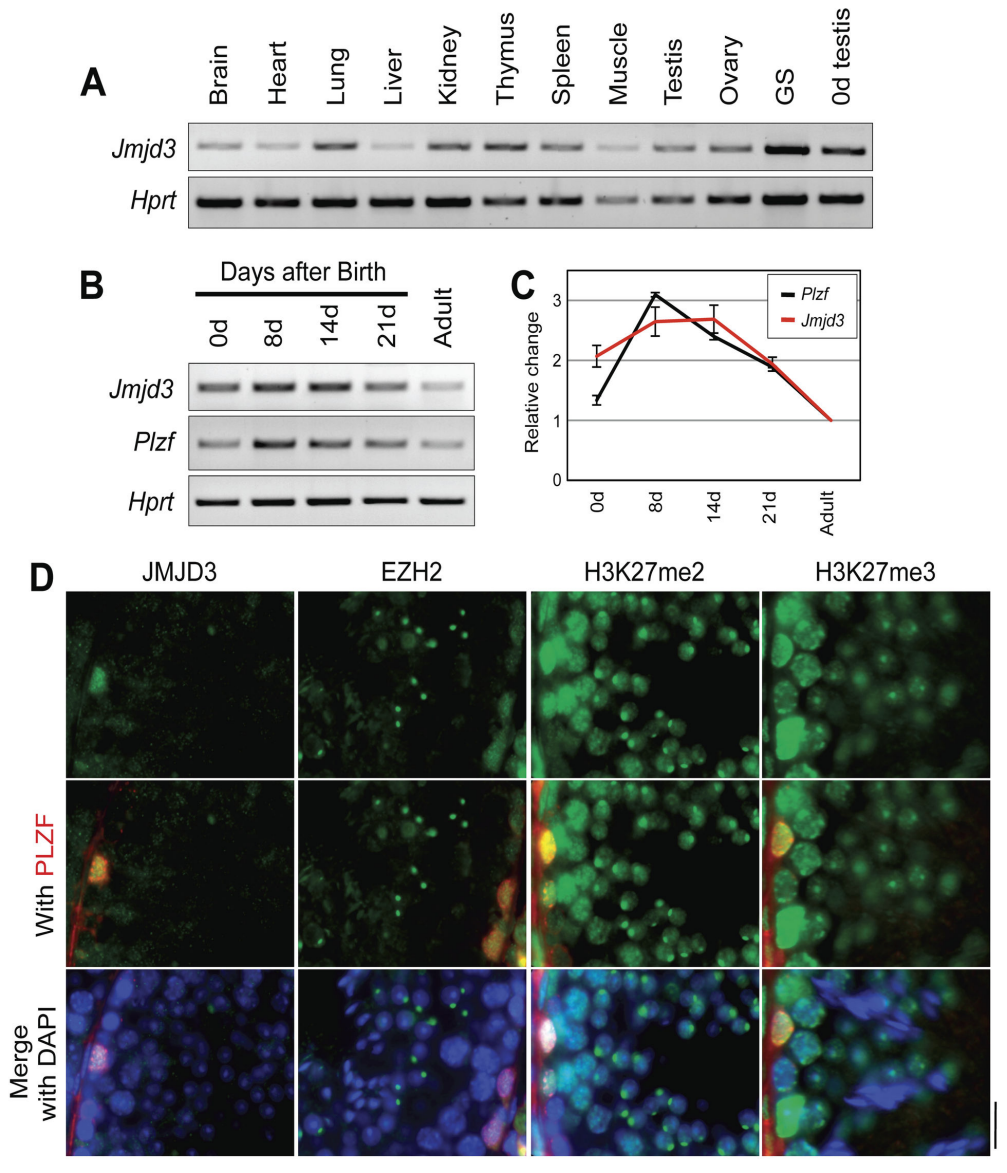
Spatiotemporal expression and testicular localization of JMJD3. Transcripts of *Jmjd3* were examined by semi-quantitative RT-PCR in multiple tissue samples (A) and during post-natal development of the testis (B). *Hprt* was used as an internal control. *Plzf* was used as a control of spermatogonial stem cell-specific gene. GS: germline stem cells. C. Relative changes of *Jmjd3* and *Plzf* during postnatal development of the testis. Relative densities of PCR products to the adult are presented after normalization with *Hprt*. Vertical bars represent the SEM of at least three experiments. D. Localization of H3K27 methylaton and its modifiers in the testis. Representative immunofluorescence images of indicated proteins with PLZF and DAPI in wild type adult testis are shown. Scale bar: 10µm.

When spatiotemporal expression patterns of *Jmjd3* were examined, *Jmjd3* was expressed in all tissues analyzed (Figure 1). Notably, *Jmjd3* was expressed higher in neonatal testis than in adult testis, and expression of *Jmjd3* was higher in cultured germline stem cells (GS cells) than neonatal testis, suggesting that *Jmjd3* expression could be preferentially enriched in undifferentiated spermatogonia (Figure 1A). The expression of *Jmjd3* in developing testis increased over the first postnatal week and decreased thereafter similar to the expression pattern of *Plzf*, an essential gene for SSC self-renewal in developing testis (Figure 1B and 1C) [27,28]. Furthermore, JMJD3 protein co-localizes with PLZF-positive undifferentiated spermatogonia in testis (Figure 1D). As shown in Figure 1D, both H3K27me3 and H3K27me2, which are targets of JMJD3 enzymatic activity, and H3K27 methyltransferase EZH2 are detected in PLZF-positive spermatogonia. A previous report indicated that specific distribution of EZH2 was in the nuclear apical region of round spermatids [29]. Even though highest localization of EZH2 was detected in nuclear apical region of round spermatids and H3K27me2 was localized in the similar region of round spermatids in our study, EZH2 was also detected in PLZF-positive spermatogonia. When JMJD3 localization in the developing testes was analyzed, JMJD3 was co-localized in PLZF-positive spermatogonia in 1-week and 3-week old testes. JMJD3 is also observed in PLZF-negative spermatogonia in 3-week old testes, suggesting that JMJD3 could function not only in undifferentiated spermatogonia but also in early stage differentiating spermatogonia (Figure 1S).

### *In vitro* effects of JMJD3 knockdown in SSCs

**Figure 2:**
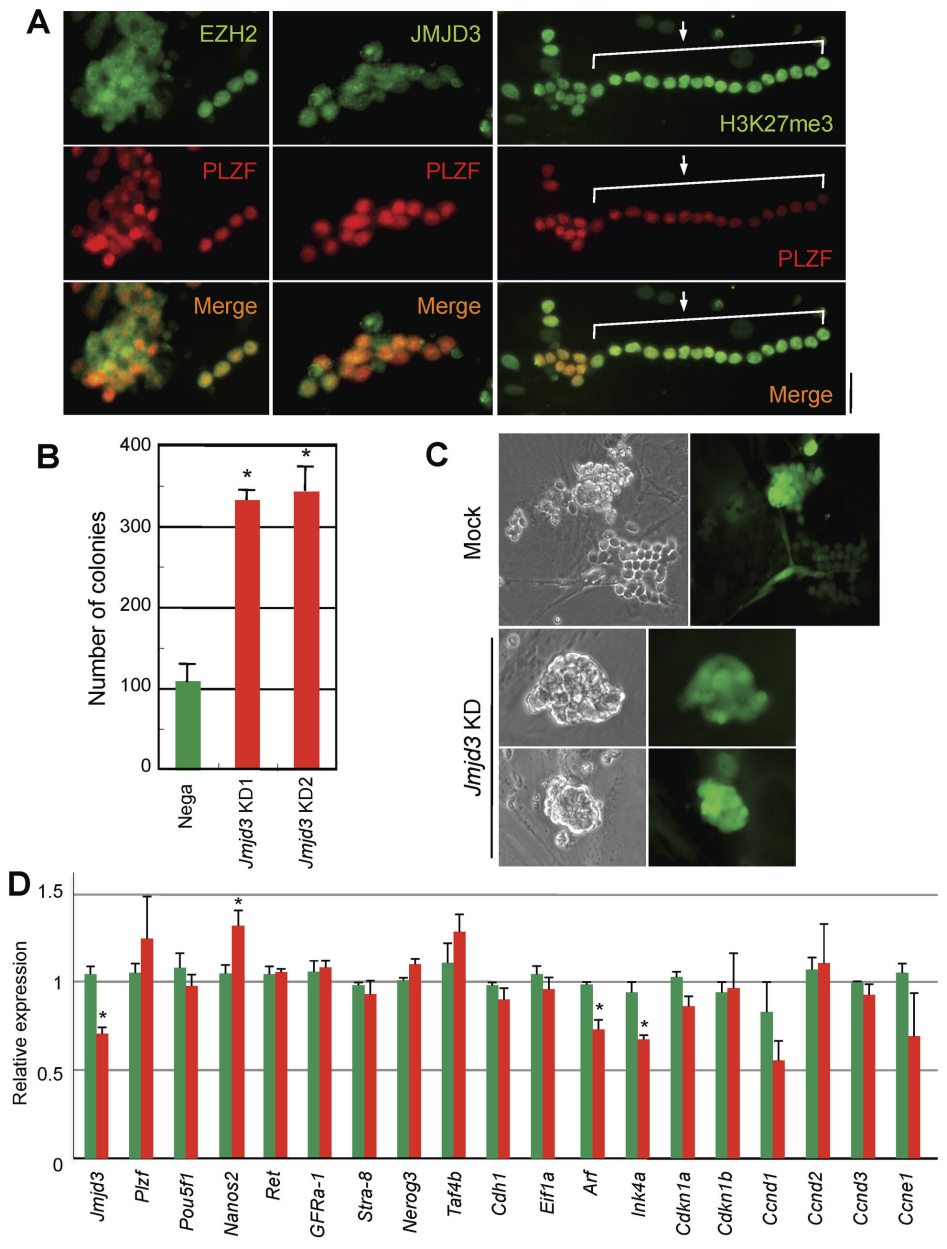
Effect of *Jmjd3* knockdown in germline stem cells. A. Immuno-localization of H3K27me3 and H3K27 modifiers in germline stem (GS) cells. Colocalization of H3K27me3, EZH2, and JMJD3 with PLZF in GS cell are shown. Notably, PLZF strongly localizes in clusters and weakly localized in chains of GS colony. Arrow and solid line indicate chains of GS cells. B. The number of colonies formed by *Jmjd3* and mock knockdown. The number of colonies at 1 week after replating of mock (green) and *Jmjd3* (red) knockdown GS cells were counted. Bars indicate mean ± se. * indicates that there is significant difference (P<0.05). C. Representative morphology of mock and JMJD3 KD GS cell colony. D. Relative expression of spermatogonial stem cell markers and cell cycle regulators in mock and JMJD3 KD GS cells. Relative expression of indicated genes are quantitatively analyzed after normalization with *Gapdh*. * indicates that there is significant difference (P<0.05).

To define the roles of JMJD3 in SSCs, we first studied GS cells *in vitro*. Both JMJD3 and EZH2 were detected in PLZF-positive undifferentiated spermatogonia (Figure 2A). H3K27me3 was weakly observed in clusters of GS cell colonies and strongly accumulated in chains of GS cell colonies, whereas PLZF was strongly localized in clusters and weakly localized in chains of GS cells, suggesting that H3K27me3 could be increased as spermatogonia processed to differentiate and/or that methylation of H3K27me3 could be slightly inhibited in undifferentiated spermatogonia and be released from the inhibition as differentiation (Figure 2A). To examine if JMJD3 was required for self-renewal or differentiation of SSCs, JMJD3 mRNA expression was reduced by shRNA-mediated knockdown (KD). When *Jmjd3* was knocked-down, the number of *Jmjd3* KD colonies formed was about three times as many as that of mock KD colonies (Figure 2B). Whereas morphology of most of the mock control KD colonies were grape-like structures, about 70% of the *Jmjd3* KD GS cells formed more compact cluster-like structures (Figure 2C). To validate the effect of *Jmjd3* KD in GS cells, expression levels of SSC markers and genes related to cell cycle regulation were analyzed. The *Jmjd3* KD effect was achieved at only 30% in our experimental systems (Figure 2D). Expression levels of p19 *Arf* and p16 *Ink4a*, which are known direct targets of JMJD3 [30,31], were decreased by *Jmjd3* KD as previously described (Figure 2D). Although expression of most genes were not significantly changed, *Nanos2*, which is restricted in its expression to undifferentiated spermatogonia [32], was significantly increased by *Jmjd3* KD. These results indicate that JMJD3 might play essential roles for differentiation of SSCs *in vivo*.

### A germ cell-specific JMJD3 conditional knockout male mouse sires offspring for a prolonged period

**Figure 3:**
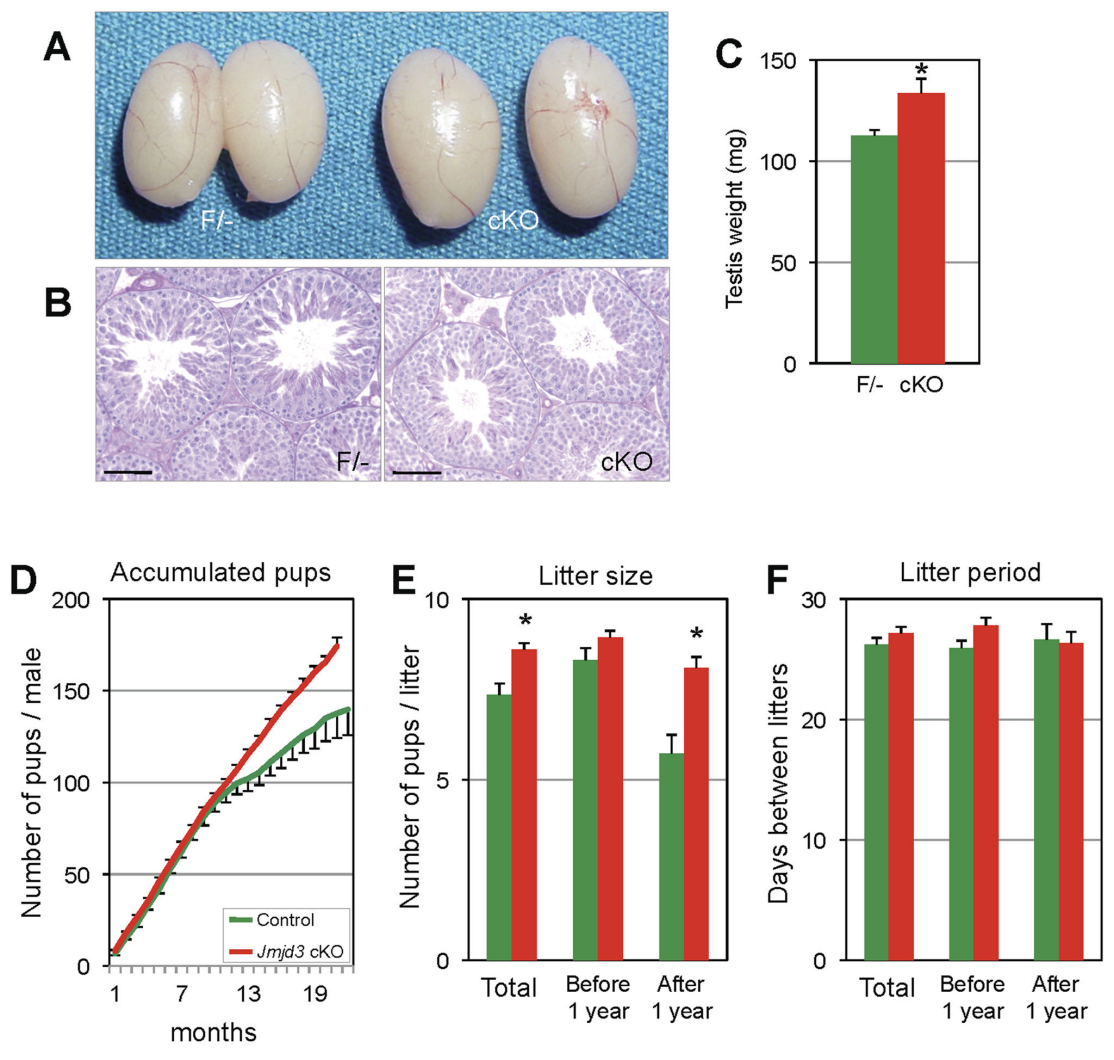
Testis phenotype and fertility analysis of JMJD3 cKO. A. Gross picture of control (F/-) and JMJD3 cKO (cKO) testes. B. Histology of control (F/-) and JMJD3 cKO (cKO) testis. Scale Bar: 50 µm. C. Testis weights of control (F/-) and JMJD3 cKO (cKO) mouse. * indicates that there is significant difference (P<0.05). D. Average number of pups produced by control (F/-) and JMJD3 cKO (cKO) males over 20 months of breeding (n=7 per genotype). E and F. Average litter sizes (E) and litter period (F) for total period, first 1 year, and after 1 year of breeding produced from control (F/-) and JMJD3 cKO (cKO) males.

To address *in vivo* functions of JMJD3, a germ cell-specific *Jmjd3* deleted mouse was generated. We designed a targeting construct to flank exons including the JmjC domain, which is a responsive domain of histone demethylase activity, with *loxP* sequences (Figure S2A). A conditional targeted allele of *Jmjd3* was generated in embryonic stem (ES) cells by homologous recombination and successful recombination was verified by Southern blot analyses using 5’ and 3’ probes (Figure S2B). A *Pgk1-Neo* cassette, which was used for selection of targeted ES cells, was removed by *Flp/Frt* recombination in mice before proceeding to other experiments because insertion of the *Pgk1-Neo* cassette results in post-neonatal lethality similar to *Jmjd3* knockout mice [20]. To analyze *in vivo* function of JMJD3 in SSCs, germ cell-specific deletion of *Jmjd3* was accomplished by crossing with *Stra8-Cre* transgenic mice, in which *Cre* was expressed in spermatogonia including SSCs starting from ^~^3 days after birth [33]. In all of the mouse experiments, we compared controls lacking Cre expression (*Jmjd3* ^*F/-*^) and JMJD3 cKO mice expressing Cre in the male germline (*Jmjd3* ^*F/-;Stra8-cre+*^). Although JMJD3 cKO males were fertile and spermatogenesis looked normal, the sizes of the adult JMJD3 cKO testes were bigger than the control testes (Figures 3A–C, and S1D). In addition, JMJD3 cKO males sired pups much longer without a change in the size of the litters, whereas litter sizes of offspring derived from controls started to decrease after 1 year of mating (Figure 3D and 3E). The rate of litter production was unchanged though (Figure 3F).

### Effects of loss of JMJD3 against H3K27 methylation and its regulators during spermatogenesis

**Figure 4:**
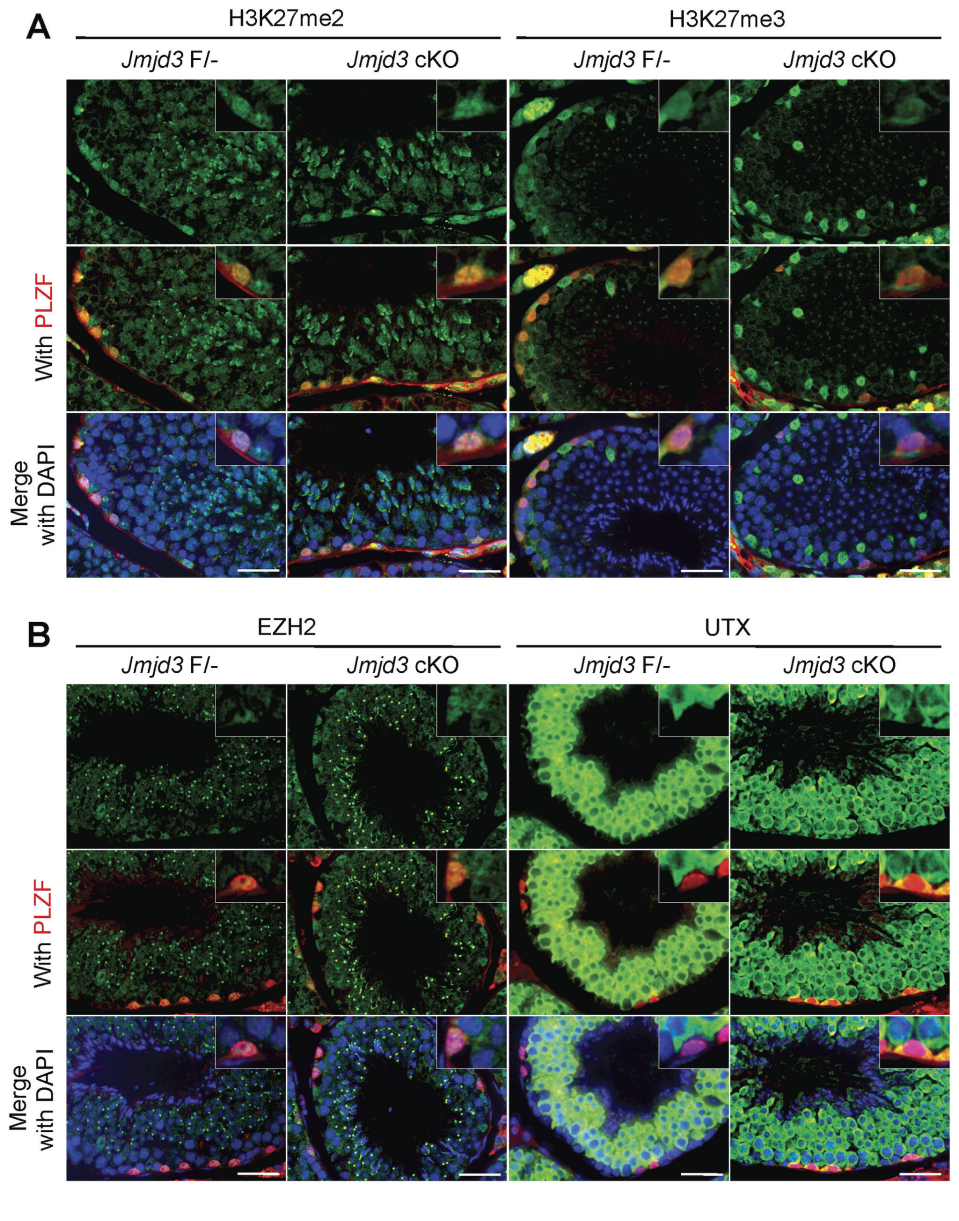
Distribution of methylated H3K27 and H3K27 modifiers in the JMJD3 cKO tsetis. A. Distribution of methylated H3K27. Immunofluorescence images of di-methylated and tri-methylated H3K27 with PLZF are shown. Insets are higher magnification images of PLZF positive spermatogonia. B. Distribution of H3K27 modifiers. Immunofluorescence images of EZH2 and UTX with PLZF are shown. Insets indicate higher magnification images of PLZF-positive spermatogonia. Scale bar: 25 µm.

To examine whether loss of JMJD3 affected global H3K27 methylation during spermatogenesis, particularly in spermatogonia, the distribution of methylated H3K27 was analyzed by immunofluorescence staining. There were no significant changes of H3K27me3 and H3K27me2 between control and JMJD3 cKO testes, suggesting that loss of JMJD3 did not affect global methylation of H3K27. Next, the distribution of H3K27 methyltransferase, EZH2, and the other H3K27 demethylase, UTX, during spermatogenesis in the absence of JMJD3 was also examined. There were no significant differences in the distribution of EZH2 between control and JMJD3 cKO. Although UTX was not detectable in control PLZF-positive spermatogonia, it localized to PLZF-positive undifferentiated spermatogonia in JMJD3 cKO testis (Figure 4), suggesting that UTX might play a functionally redundant role with JMJD3 in the JMJD3 cKO spermatogonia.

### Characteristics of JMJD3 null spermatogonia

**Figure 5:**
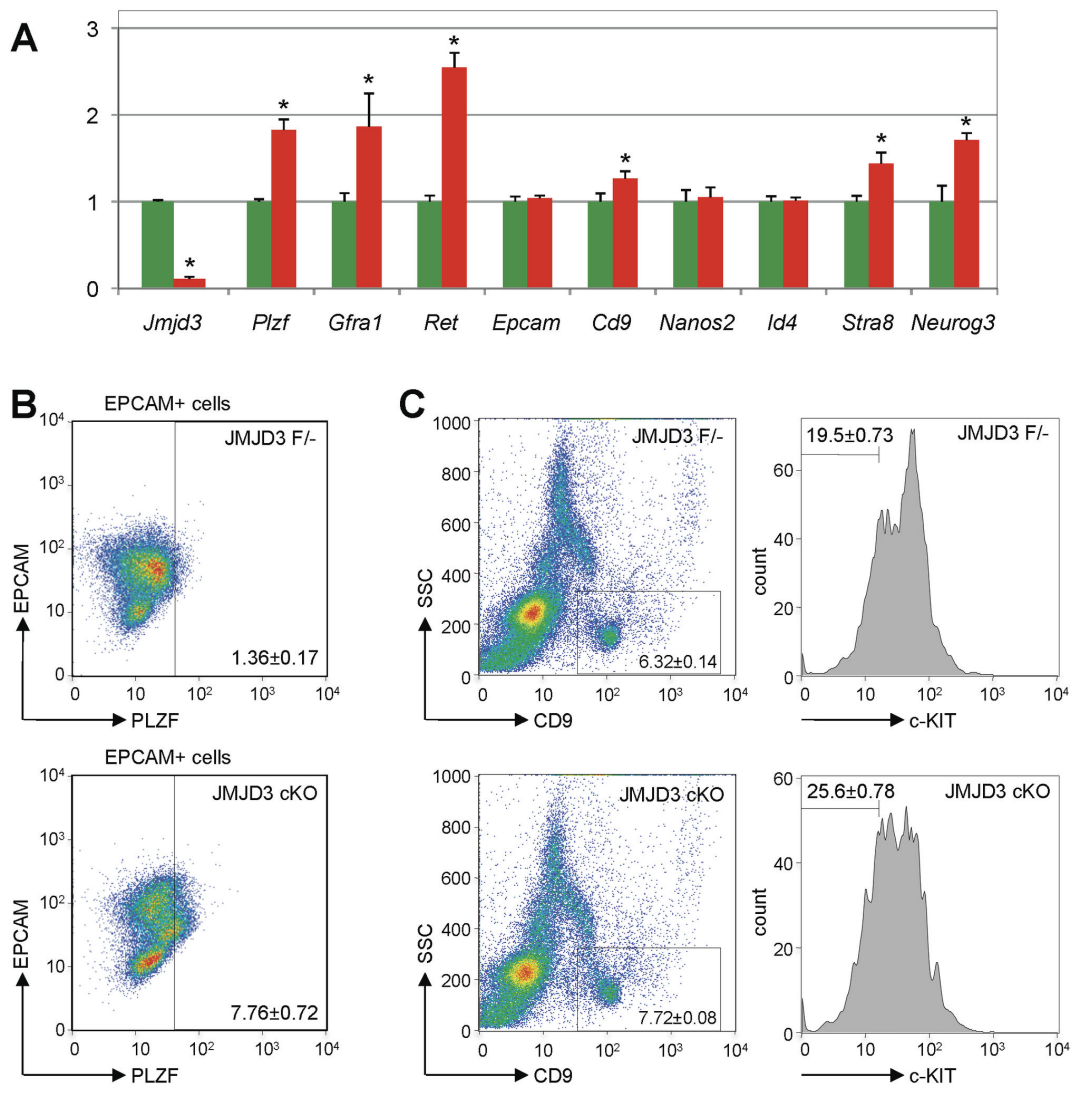
Characteristics of JMJD3 null undifferentiated spermatogonia. A. Relative expressions of SSC markers in control (F/-) and JMJD3 cKO (cKO) EPCAM-positive testicular cells were quantitatively analyzed. All of the expression levels were normalized to *Gapdh* expression. * indicates that there is significant difference (P<0.05). B. Flow cytometric analysis of EPCAM and intracellular staining of PLZF in the EPCAM-positive JMJD3 F/- (upper) and JMJD3 cKO (lower) spermatogonia. Ratio of PLZF positive cells is significantly different (P<0.01). C. Flow cytometric analysis of side scatter and CD9 in the JMJD3 F/- and the JMJD3 cKO testicular cell (Left panels). Ratio of side scatter-low and CD9+ cells is statistically different (P<0.001). Right panels: KIT expression in the side scatter-low and CD9+ JMJD3 F/- and JMJD3 cKO cells. Ratio of KIT-negative cells is significantly different (P<0.001).

To determine the effects of JMJD3 ablation in SSCs, expression of genes known to be transcriptionally active in SSC were analyzed in EPCAM-positive spermatogonia, in which EPCAM-positive cells were enriched using magnetic activated cell sorting (MACS) [34]. First, reduction of JMJD3 was confirmed in more than 90% of EPCAM-positive spermatogonia (Figure 5A). JMJD3 positive protein signal could not be detected even in the EpCAM-selected spermatogonia, even if PLZF-positive spermatogonia are enriched in the fraction, probably because JMJD3-positive spermatogonia may be rare in EpCAM-selected cell fraction and expression of JMJD3 may not be high enough in each spermatogonia. Many SSC markers (*Plzf, Gfra1, Ret, Cd9, Stra8*, and *Neurog3*) were significantly increased in JMJD3 null spermatogonia, suggesting that undifferentiated spermatogonia are increased in the EPCAM-positive spermatogenic cell population (Figure 5A). However, *Nanos2* was not significantly changed between control and JMJD3 cKO testes (Figure 5A) in contrast to the results from the *in vitro* experiments using JMJD3 knockdown GS cells (Figure 2D).

To characterize the population of undifferentiated spermatogonia in JMJD3 cKO testes, EPCAM-positive spermatogonia enriched by MACS and total testicular cells were subjected to flow cytometric analyses. According to a previous report, SSCs are enriched in the EPCAM-dim and CD9-positive fraction [34]. When EPCAM-positive cells were analyzed by combination of EPCAM staining with intracellular staining of PLZF, PLZF-positive cells were dramatically increased in the JMJD3 cKO EPCAM-dim cell population as EPCAM-dim cell population was exhibited to move to the PLZF-positive direction as shown in Figure 5B. Since SSCs have characteristics of side scatter signal low, CD9-positive, and KIT-negative, combination among side scatter, CD9, and KIT expression in testicular cells were analyzed. Side scatter-low and CD9-positive cells were significantly increased in JMJD3 cKO testis (P<0.001: Figure 5C). Moreover, KIT negative cells were significantly increased in JMJD3 cKO side scatter-low/CD9-positive cell population (P<0.001; Figure 5C). These results suggest that undifferentiated spermatogonia are significantly increased in testis by JMJD3 ablation.

### Increase of A_s_ spermatogonia in JMJD3 cKO seminiferous tubules

**Figure 6:**
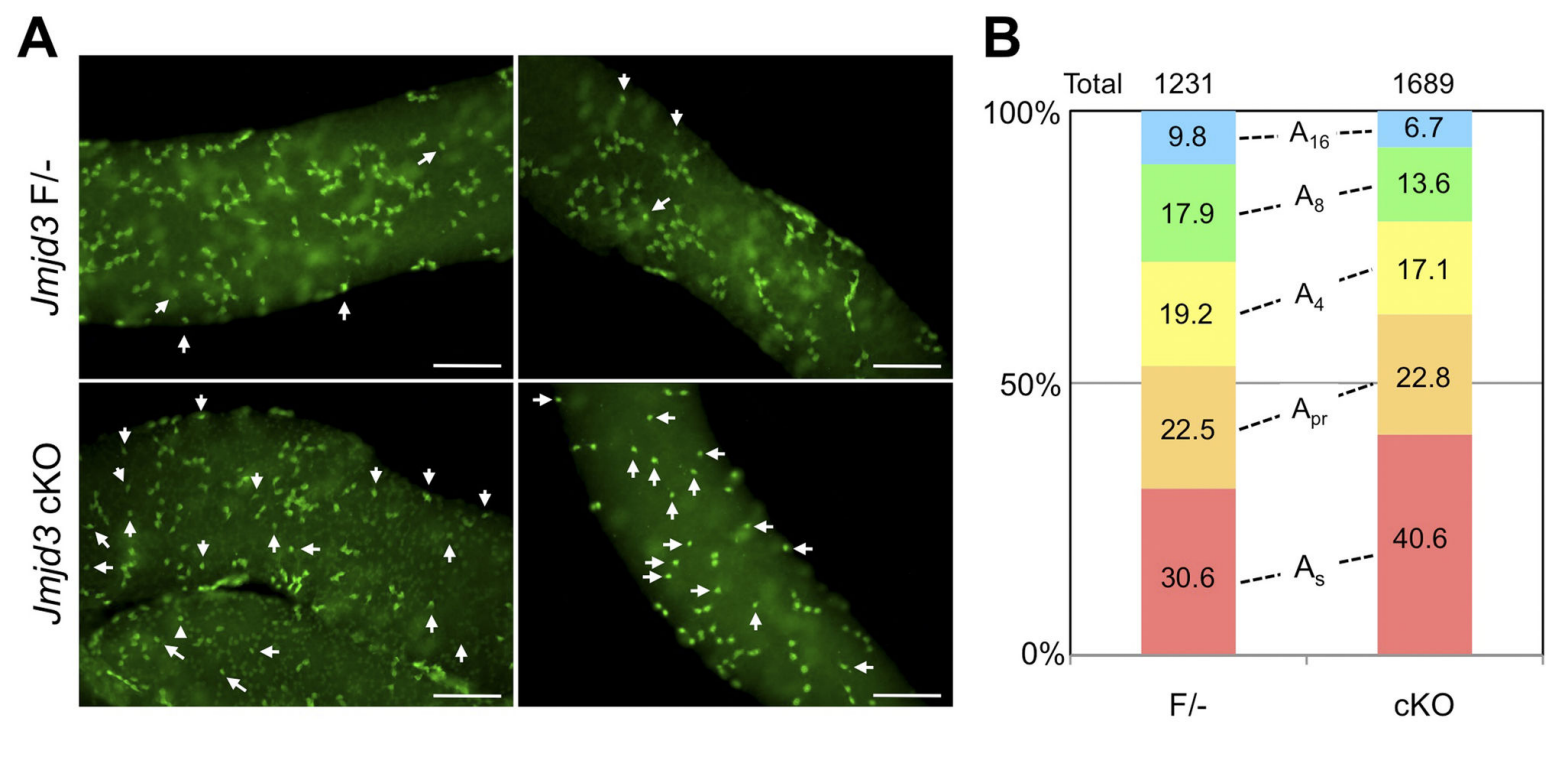
Effect of JMJD3 ablation to the spermatogonial chain formation. A. Whole mount staining of seminiferous tubules for PLZF (green). Arrows indicate A_s_ spermatogonia. Scale bar: 100 µm. B. The ratio of PLZF-positive spermatogonial cyst in control (F/-) and JMJD3 cKO (cKO) seminiferous tubules. Total number of colonies counted in the experiment are shown above the graph.

To further examine if loss of JMJD3 affected SSC behavior, spermatogonial chain formation was visualized by whole mount immunostaining of PLZF in seminiferous tubules. Only a few A_s_ spermatognia were detected in control tubules, whereas unexpectedly many A_s_ spermatogonia exist in JMJD3 cKO tubules (Figure 6). When more than one thousand PLZF-positive spermatogonial chain colonies were counted, 30% of the colonies were A_s_ in control tubules, whereas 40% of colonies existed as A_s_ in JMJD3 cKO tubules (Figure 6B). The ratio of A_pr_ colonies in the JMJD3 cKO tubules are equivalent to the control tubules. The ratio of A_al_ colonies, which includes A_4_, A_8_, and A_16_ spermatogonia, are decreased in JMJD3 cKO tubules. These results suggest that many undifferentiated spermatogonia could be maintained for a longer period in JMJD3 cKO testes or undifferentiated spermatogonia could be increased and maintained by frequent symmetric division of SSC.

### Frequent spermatogonial dedifferentiation by JMJD3 ablation

**Figure 7:**
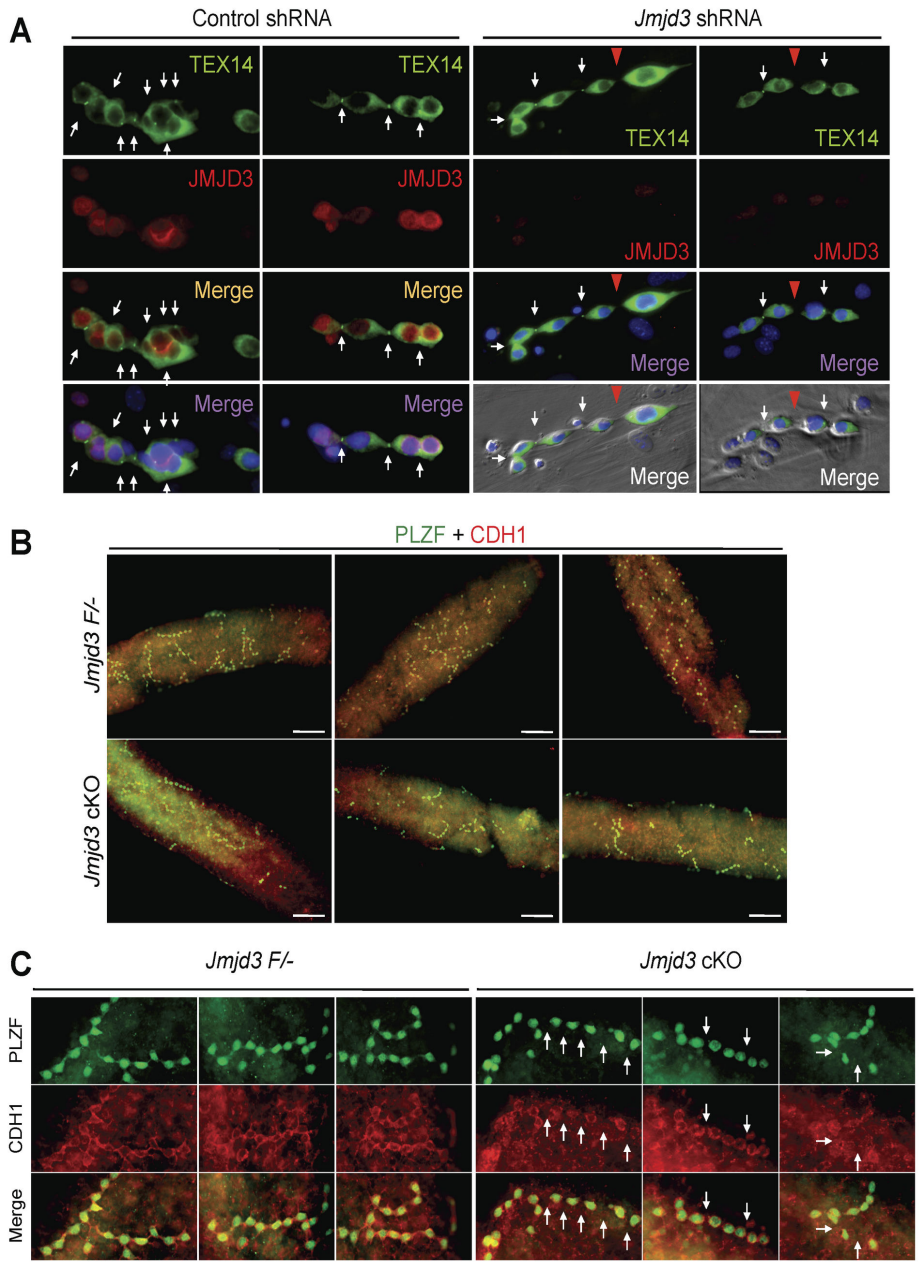
Disruption of intercellular bridges by loss of JMJD3. A. Immuno-staining of JMJD3 (red) and TEX14 (green) in mock KD and JMJD3 KD GS cells. Cells were fixed and stained at 5 days after shRNA induction. White arrows indicate TEX14 positive intercellular bridges and red arrowhead indicates connective region of two adjacent cells without intercellular bridge. B and C. Whole mount staining of PLZF (green) and CDH1 (red) in the JMJD3 F/- and the JMJD3 cKO seminiferous tubules. Low magnification images (B) and high magnification images of individual colonies (C) are shown. Scale bar: 100 µm.

When intercellular bridge formation was analyzed in control colonies, TEX14-positive intercellular bridges were detected between all cells in individual colonies (Figure 7A). In contrast, TEX14 signals disappeared from some connecting regions of cells in the individual JMJD3 KD colonies (Figure 7A). As shown, one cell can separate from the colony and cytoplasmic TEX14 signal in the cell is stronger than other cells in the colony (Figure 7A), suggesting that the separated cells behaved as a single spermatogonia. To verify whether separation of cells from growing and differentiating colony by disruption of intercellular bridge occurred *in vivo*, spermatogonial chain formation in seminiferous tubules was analyzed by whole mount staining of TEX14. However, TEX14-positive intercellular bridges were unfortunately undetectable in whole mount staining, although stronger cytoplasmic signals of TEX14 were detectable in PLZF-positive spermatogonia (Figure S3). Therefore, spermatogonial chains in seminiferous tubule were visualized by a combination of immunofluorescence analysis of PLZF and CDH1 (E-cadherin), which is specifically expressed in undifferentiated spermatogonia [35]. In control seminiferous tubules, CDH1 connects neighboring cells in the colonies, and cell morphology of individual cells in the colony are observed to be stretched (Figure 7B upper panels and Figure 7C left panels). In contrast, immunofluorescence analysis of CDH1 showed that some cells in JMJD3 cKO colonies were separated from neighboring cells, and the cells were round shaped (Figure 7B lower panels and Figure 7C right panels). This round shaped morphology of the spermatogonia was also observed in TEX14 null spermatogonia lacking intercellular bridges [3]. Notably, some cells appeared to be starting to separate from the colony (Figure 7C right panels). These results, along with the observation of intercellular bridge disruption in the *in vitro* experiments, suggest that the frequency of random and stochastic fragmentation of spermatogonial cyst was increased by loss of JMJD3. Finally, when expression levels of genes related to intercellular bridge formation were analyzed [36], there were no significant changes between mock and JMJD3 KD GS cells (Figure S4). Our results suggest that the increase in fragmentation of spermatogonial cysts by lack of JMJD3 was not caused by defects in intercellular bridge formation machinery and there could be another mechanism that regulates the destruction of intercellular bridge for fragmentation of spermatogonial cysts.

## Discussion

In this report, we have first analyzed expression and localization of methylated H3K27 and its modifiers (JMJD3, EZH2, and UTX). Localization of methylated H3K27 was not identical with a previous report. H3K27me3 is highly localized in Sertoli cells and form foci at the center of the nucleus of round spermatids in our studies but not in a previous report [21]. In addition, H3K27me2 localizes at the apical region of round spermatids near the acrosome, but not in the previous report [21]. These differences could be caused by differences in ages of the mouse testes used in the experiments: the other study used juvenile mouse testes to examine the distribution of histone methylation, whereas we used adult mouse testes (16-20 weeks of age). Since H3K27me3 accumulates in terminally differentiated cells as previously described, the highest accumulation of H3K27me3 in Sertoli cells in our results is likely because the Sertoli cells are terminally differentiated in adult testes but not in juvenile testes [37].

When *Jmjd3* expression is reduced in GS cells in culture, the number of colonies increases and the morphology of most colonies was cluster-like similar to ES cells but not grape- or chain-like as observed for wild-type GS cells. Moreover, expression of *Nanos2* was significantly upregulated in *Jmjd3* KD GS cells, although knockdown efficiency of *Jmjd3* was achieved at just about 30%. These results suggest that JMJD3 was important for the differentiation of SSCs but not self-renewal of SSCs. However, germ cell-specific JMJD3 null males are fertile and spermatogenesis looked normal, indicating that JMJD3 was not essential for differentiation of SSCs. JMJD3 cKO males show larger testis size compared to controls and prolonged fertility likely secondary to enhanced self-renewal of SSCs. In support of this, JMJD3 KD GS cells showed enhanced self-renewal activity as described above. There is an apparent difference between the *in vitro* experiments and the phenotype of JMJD3 cKO mice. This difference can be explained by detailed analyses of behavior and characteristics of JMJD3 cKO SSCs or SSC enriched cell population. Cells that had SSC characteristics increased in the JMJD3 cKO testis. Expression levels of SSC marker genes are increased in SSC enriched fractions of JMJD3 cKO testes. Moreover, we observed apparent fragmentation of spermatogonial cysts, which may mean that differentiating and growing spermatogonia could revert to a primitive stage of spermatogonia such as A_s_ spermatogonia. Spermatogonia can dedifferentiate under stress conditions and can be continuously replaced[24-26]. If JMJD3 null differentiating spermatogonia can revert to an undifferentiated state, self-renewal activity of *Jmjd3* null SSCs could be enhanced at a glance. If dedifferentiation of spermatogonia is considered, the number of SSC colonies formed *in vitro* can increase after a split of SSCs, and the SSC compartment can be maintained at a high level even if individual SSCs have reduced self-renewal activity. Moreover, differentiating spermatogonia do not need to dedifferentiate into SSCs. As long as they dedifferentiate to an undifferentiated stage such as transit amplifying cells or A_pr_ spermatogonia but not stem cells, spermatogonia can re-start or resume the differentiation cycle. Other experiments such as SSC transplantation, live imaging, and lineage tracing using the JMJD3 cKO mice will be useful to prove enhanced dedifferentiation of differentiating spermatogonia by JMJD3 deletion.

Clusters of SSC colonies could represent a more undifferentiated state compared to chains of colonies because PLZF accumulates in clusters compared to chains of GS cells. H3K27me3 exists but is not high in clusters of colonies, whereas H3K27me3 starts to accumulate in chains of colonies. The localization of H3K27me3 in GS cells suggests that JMJD3 may function more in an undifferentiated state than in a differentiating state. Alternatively, the demethylation activity of JMJD3 might not change between undifferentiated and differentiated SSCs, whereas EZH2 activity might increase as SSCs differentiate. Of course, GS cells in culture may not be identical to SSCs *in vivo*, because GS cells in culture are free to move and divide, and their proliferation could accelerate *in vitro*, whereas SSCs *in vivo* are surrounded by Sertoli cells in the postnatal testes. In any case, some specific targets of JMJD3 could regulate SSC maintenance because JMJD3 is abundant in PLZF-positive spermatogonia and H3K27me3 is a mark of repression of transcription. In JMJD3 null differentiating spermatogonia, targets of JMJD3 could remain and attempt to keep cells in an undifferentiated state. Alternatively, there might exist a specific gene that regulates turnover of differentiating spermatogonia into an undifferentiated state, and this gene might regulate JMJD3 null differentiating spermatogonia. Further experiments such as ChIP-seq to identify targets of JMJD3 will be required to understand the molecular mechanisms underlying enhanced fragmentation of spermatogonial cysts in JMJD3 null testes.

Expression of *Nanos2* was not significantly changed in the *Jmjd3* null EPCAM-positive cell fraction, although *Nanos2* expression is increased in JMJD3 KD GS cells. Furthermore, some SSC-related genes (*Plzf, Gfra1, Ret, Stra8, Neurog3*) are upregulated in JMJD3 null EPCAM-positive spermatogonia, whereas they are not significantly altered in JMJD3 KD cells. A reason for the differences in gene expression between those two groups may be efficiency of our knockdown experiments, which was just about 30%, and the impurity of the EPCAM-positive testicular cell fraction. If we could achieve knockdown efficiency over 90%, other SSC markers may change significantly. NANOS2 expression is restricted in the primitive stage of undifferentiated spermatogonia such as A_s_ and A_pr_ spermatogonia. The ratio of A_s_ was increased about 10%, and A_pr_ was not changed in PLZF-positive colonies by loss of JMJD3. Ratios of the number of A_s_ and A_pr_ in total number of PLZF-positive spermatogonia was 6.7% and 9.9% in control and 11.0% and 12.0% in JMJD3 cKO, respectively. This was a 1.4-fold increase. Moreover, PLZF-positive spermatogonia are not the majority of EPCAM-positive testicular cells. This may explain why we could not obtain significant differences in *Nanos2* expression in the EPCAM-positive cell fraction.

Expression of *Gfra1* was significantly increased in the JMJD3 null EPCAM-positive cell fraction, although *Gfra1* expression is limited to the primitive stage of spermatogonia such like *Nanos2*. GFRA1 is a receptor for GDNF, which is an essential growth factor for SSCs self-renewal [38,39]. Upregulation of *Gfra1* in JMJD3 null EPCAM+ cells may cause an increased number of SSCs in culture. Alternatively, expression of *Gfra1* in each SSCs in culture may be enhanced to receive excess amount of GDNF because GDNF is rich in culture condition but limited *in vivo* environment. Improvement of experimental conditions such as enhancement of knockdown efficiency and collection of pure SSCs using other marker(s) of SSCs will be required for understanding the characteristics of JMJD3 null SSCs, although these experiments are still limited at the present time.

Although expression of JMJD3 in human testes has not been identified, JMJD3 could be expressed in spermatogonia in the primate spermatogenesis. Formation of intercellular bridges as well as chains of spermatogonia in the primate spermatogenesis has to be characterized well to apply rodent results. However, there are TEX14-positive intercellular bridges in primate spermatogenesis and primate spermatogonial cysts could be fragmented by abscission of intercellular bridges. Therefore, JMJD3 may regulate the fragmentation of spermatogonial cysts from A_pale_ spermatogonia to A_dark_ spermatogonia in the primate. Treatment of infertile patients could be improved by inhibition of JMJD3 to expand the number of spermatogonia *in vivo* and/or *in vitro*, because inhibition of JMJD3 can be easily achieved by small molecule treatment [40].

## Methods

### Semi-quantitative and quantitative RT-PCR

Total RNA from mouse adult tissue samples and cultured and isolated cell samples were extracted using RNeasy mini and micro kit (QIAGEN), respectively, according to the manufacturer’s instruction and reverse transcribed using Superscript III reverse transcriptase (Invitrogen) and an oligo-dT primer (Invitrogen). For semi-quantitative RT-PCR, PCR amplifications were performed using *Taq* DNA polymerase and visualized by gel electrophoresis. For quantitative PCR, PCR amplifications were performed using SYBR Green PCR Master Mix (ABI) and analyzed by the ABI 7500 sequence detection system. The primer sequences used in the experiments are listed on Supplementary Table 1.

### Histology and Immunofluorescence

Testes were fixed in 4% paraformaldehyde or Bouin’s fixative overnight, followed by paraffin embedding. Tissue embedding was performed by the Department of Pathology Histology Core (Baylor College of Medicine). Five micrometer sections were stained with periodic acid-Schiff reagent and counterstained with hematoxylin. For immunofluorescence of tissue sections, paraformaldehyde fixed sections were retrieved by microwave, and then incubated with primary antibodies overnight at 4^°^ C, followed by Alexa 488 and Alexa 594 conjugated secondary antibodies (Invitrogen) for 1 hour at room temperature. Fluorescent sections were mounted with VECTASHIELD containing DAPI (VECTOR Laboratories). For cultured cells, cells were fixed with 2% parafomaldehyde for 10 min and permeabilized with 0.5% NP-40 for 15 min. After incubation with primary antibodies for 2 hours at room temperature, signals were visualized by Alexa 488 and Alexa 594 conjugated secondary antibodies (Invitrogen). Primary antibodies used in the experiments were as follows: rabbit anti-JMJD3, rabbit anti-EZH2, and rabbit anti-UTX (Abcam), rabbit anti-H3K27me3 and rabbit anti-H3K27me2 (Upstate Biotechnology, now EMD Millipore), mouse anti-PLZF (clone 2A9; Calbiochem, now Merck Millipore).

### Cell Culture and transfection

Culture and establishment of spermatogonial stem cells were performed as described previously using Stempro34 (Invitrogen) based media [3], [41]. For virus transfection, a shRNA against JMJD3 were subcloned into pLKO.3G vector. Virus particles were produced by transient transfection of viral plasmid with pMDLg/pRRE, pRSV-Rev, and pCMV-VSVG into 293T cells. SSCs were infected with lentivirus particles for 3 days in the presence of protamine sulfate. Five days after infection, cells were re-plated onto mitomycin-C treated mouse embryonic fibroblast and number of colonies was counted at 1 week after re-plating. Targeting sequences used for mouse *Jmjd3* shRNA were 5’-GCTGGATGAATCCATTCGGAA-3’ (*Jmjd3*-1) and 5’-CCTGTATATGTCTCTTGTTTA-3’ (*Jmjd3*-2).

### Generation of germ cell specific JMJD3 null mice and fertility analysis

A targeting construct was generated using a recombineering strategy [42], [43]. Briefly, 10.5 kb of genomic region of *Jmjd3* was retrieved from BAC bMQ73n09 (Welcome Trust Sanger Institute) and inserted into pBluescript SK containing diphtheria toxin A for negative selection (pDTA.3 kindly provided by Dr. Pumin Zhang). A *loxP* sequence was inserted between exon 20 and 21, and an *Frt - Pgk1-Neo – Frt - loxP* cassette was inserted between exon 13 and 14 to frank exons containing *JmjC* domain with *loxP* sequences. The linearized targeting construct was electroporated into E14Tg2A embryonic stem (ES) cells, which are derived from 129Ola strain mice. ES cell clones were selected in ES cell culture media supplemented with 0.18 mg/ml G418. Targeted clones were screened by Southern blot analysis using 5’ and 3’ probes. Three of the correctly targeted clones were expanded and injected into C57BL/6J blastocysts. Chimeric males were bred to C57BL/6J females to obtain heterozygous *Jmjd3* floxed mice (*Jmjd3* ^*Floxed/-*^), followed by mating with Flp expressing mice to remove *Pgk1-Neo* cassette (*Jmjd3* ^*F/-*^) and by mating with Stra8-Cre mice [33] (kindly provided by Dr. Robert E. Braun, Jackson Laboratory) to generate *Jmjd3* ^*+/-; Stra8-Cre+*^ mice. Germline specific mutant mice (JMJD3 cKO; *Jmjd3* ^*F/-;Stra8-Cre+*^) were produced by crossing male *Jmjd3* ^*+/-; Stra8-cre+*^ and female *Jmjd3* ^*F/F*^ mice. Mice were genotyped by genomic PCR analysis using the following primers: WT/KO forward, GCGAGAGACCTGAGGCATGA; WT reverse, CTCGCCTCCACCAGAGTCTT; KO reverse, AGGGGGAAGAGCTTGCACAC. For fertility analysis, six-week-old control (*Jmjd3* ^*F/-;Stra8-Cre-*^) and JMJD3 cKO (*Jmjd3* ^*F/-;Stra8-Cre+*^) male littermates were individually bred to WT females. After 1 year from initiation of mating, females were replaced to 6-week-old young females. The number of litters and pups born per litter were monitored over an 18-month period. All mouse experiments were performed on a C57BL/6J: 129Ola hybrid background in accordance with protocols approved by the Institutional Care and Use Committee of Baylor College of Medicine.

### Magnetic activated cell sorting and flow cytometry

After removal of tunica, isolated testes were treated with a two-step enzymatic digestion with 1 mg/ml type IV collagenase (Sigma) and 0.25% trypsin in the presense of 0.7 mg/ml DNase (Sigma), followed by resuspension in PBS containing 3% FBS for subsequent staining and analysis. Suspended testicular cells were incubated with rat anti-EPCAM (CD326) antibodies (eBiosciences) on ice for 30 min, followed with anti rat IgG microbeads (Miltenyi Biotec) on ice for 20 min. EPCAM-positive cells were separated by MACS cell separation system (Miltenyi Biotec) according to the manufacturer’s instructions. Total testicular cells were incubated with Alexa488 conjugated anti-CD9 and allophycocyanin (APC) conjugated anti-c-KIT on ice for 30 min. EPCAM-positive cells were treated with FoxP3/transcription factor staining kits (eBiosciences) according to manufacturer’s instructions. Permeabilized cells were incubated with phycoerythrin (PE) conjugated anti-EPCAM (eBiosciences) and Alexa 488 conjugated anti-PLZF antibodies, which anti-PLZF antibodies (2A9; EMD Millipore) was directly conjugated to Alexa 488 using Alexa Fluor 488 antibody labeling kit (Invitrogen) according to the manufacturer’s instruction. Stained cells were typically analyzed on a FACSCanto II (BD Biosciences) and data were analyzed by FlowJo software (Tree Star).

### Whole mount immunostaining

After removal of tunica, seminiferous tubules were dispersed by treatment with 1mg/ml type IV collagenase (Sigma) in Hank’s balanced salt solution (HBSS; Invitrogen) at 37 ^°^C for 10 min with gentle agitation. Tubules were washed with HBSS to remove interstitial cells and fixed in 4% paraformaldehyde for 4 hours on ice. Tubules were dehydrated through increasing concentration of ice-cold methanol (25, 50, 75, 100% in PBS), following incubation with 0.2% NP-40 in PBS for 30 min. After rehydration in PBS containing 0.1% Tween-20 (PBS-T), non specific antibody binding was blocked with 1% bovine serum albumin (BSA; Sigma) and 5% normal donkey serum (Jackson Immunoresearch) in PBS-T for 1 hour. Tubules were incubated with primary antibodies diluted in blocking solution overnight at 4^°^ C. After washing with PBS-T, tubules were incubated with secondary antibodies for 2 hours at room temperature. After washing with PBS-T, specimens were mounted in VECTASHIELD (VECTOR Laboratories). Primary antibodies used were as follows: rabbit polyclonal anti-PLZF (Santa Cruz Biotechnology), mouse monoclonal anti-PLZF (2A9; Calbiochem), rat monoclonal anti-CDH1 (ECCD-2; EMD Millipore), goat polyclonal anti-TEX14, Alexa 488 or 594 conjugated donkey anti-rabbit, anti-rat, anti-mouse, anti-goat antibodies.

### Statistical Analysis

Data are presented as the mean ± SEM. Statistical significance was determined by Student’s *t*-test. The ratio of PLZF-positive spermatogonial cyst was analyzed using ANOVA followed by Tukey-HSD. P<0.05 was considered statistically significant.

## Supporting Information

Figure S1 **Localization of JMJD3 in developing testes.** Representative Immunofluorescence images of JMJD3 (green) with PLZF (red) in 1- and 3-week old testis are shown.
(TIFF)

Figure S2 **Generation of *Jmjd3* targeted allele.** A. Strategy of targeting conditional allele of the *Jmjd3* gene. An *Frt - PGK1-Neo – Frt - loxP* cassette and a *loxP* sequence were inserted between exons 13 and 14 and between exons 20 and 21, respectively. DTA, diphtheria toxin fragment A. B. Southern blot analyses using 5’ (left panel) and 3’ (right panel) external probes. XbaI (Left) and BamHI (Right) digested ES cell DNA were hybridized with probes that detect a 16.0 kb WT and 8.0 kb targeted alleles at the 5’ end (Left), or a 13.4 kb WT and a 6.2 kb targeted alleles at the 3’ end (Right). C. Confirmation of the *Jmjd3* null allele by genomic PCR. PCR using primers spanning floxed region and Stra8-Cre allele are shown. *Pgk1-Neo* cassette was removed by *Flp/Frt* recombination because insertion of the cassette resulted in post-neonatal lethality similar to *Jmjd3* knockout mice. D. Histology of control (F/-) and JMJD3 cKO (cKO) testis at 3-week of age. Scale bar: 50 µm.
(TIFF)

Figure S3 **Visualization of spermatogonial chain with TEX14 and PLZF.** Whole mount immuno-staining of seminiferous tubules for TEX14 and PLZF. Representative pictures of TEX14 and PLZF staining are shown. Scale bar: 50 µm.
(TIFF)

Figure S4 **Effect of loss of JMJD3 for intercellular bridge formation.** Relative expression of genes related for intercellular bridge formation (*Tex14*, *Cep55*, *Alix*, *Tsg101*, *Alix*, *Kif23*, *Plk1*) are shown. All expression levels were normalized to *Gapdh* expression.
(TIFF)

Table S1 Primer sequences used in the study.
(PDF)
