# Supplementary figures and images for: H3K27 Demethylase, JMJD3, Regulates Fragmentation of Spermatogonial Cysts
Source: PLoS One. 2013 Aug 15;8(8):e72689. doi: 10.1371/journal.pone.0072689 (PMC3744460; doi:10.1371/journal.pone.0072689)

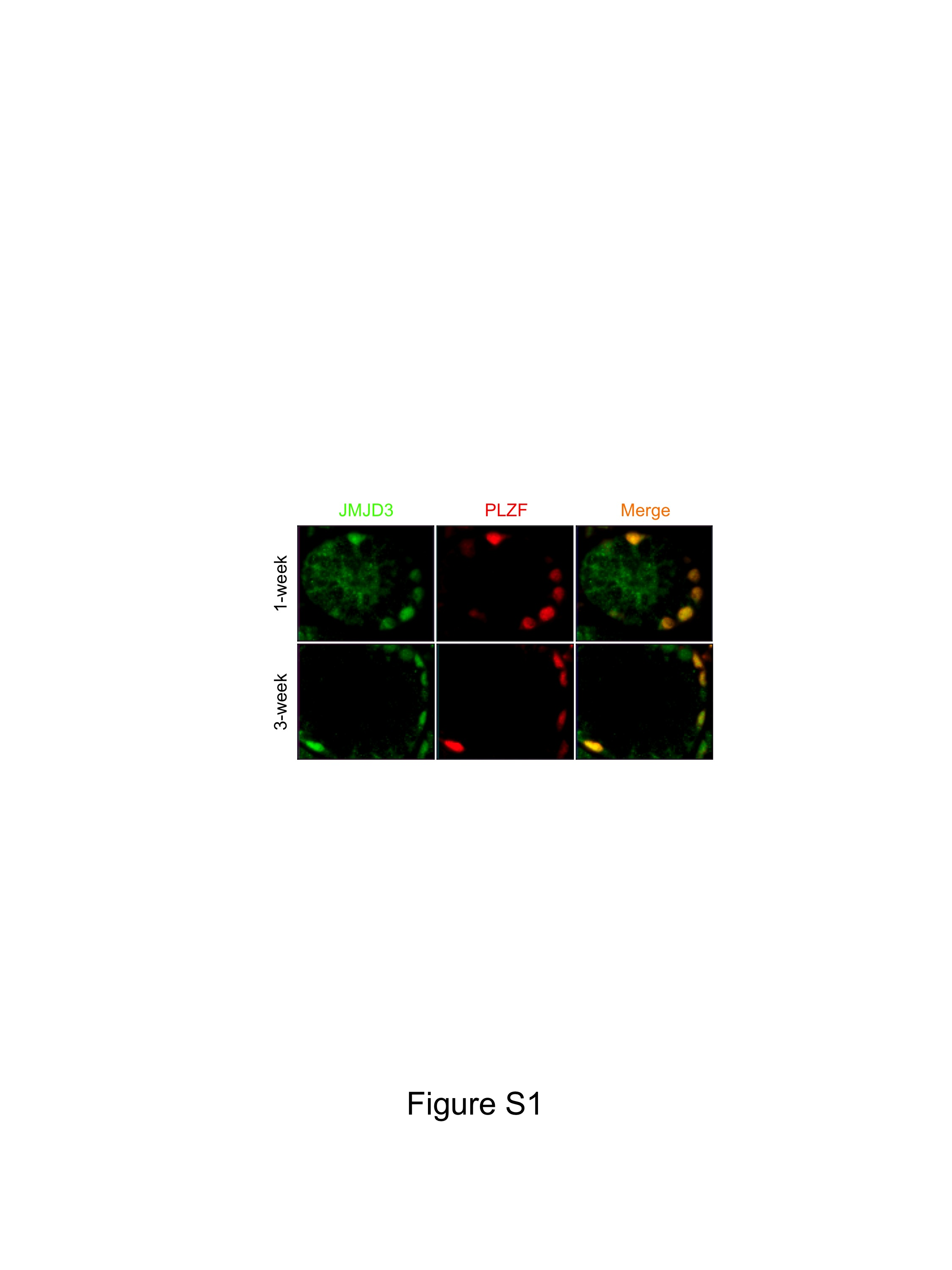

Supplement: Figure S1 — Localization of JMJD3 in developing testes. Representative Immunofluorescence images of JMJD3 (green) with PLZF (red) in 1- and 3-week old testis are shown. (TIFF) [file pone.0072689.s001.tiff]

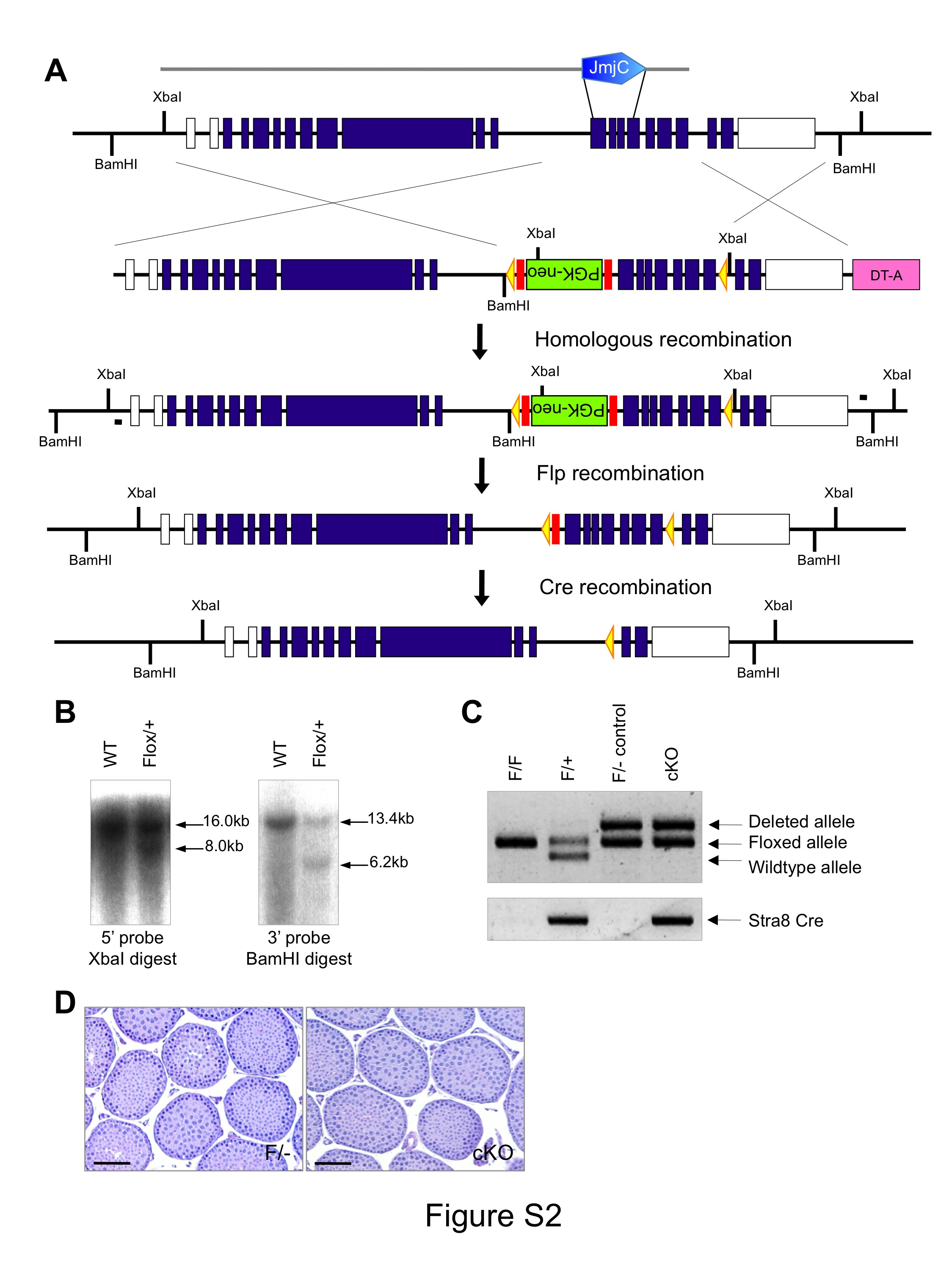

Supplement: Figure S2 — Generation of Jmjd3 targeted allele. A. Strategy of targeting conditional allele of the Jmjd3 gene. An Frt - PGK1-Neo – Frt - loxP cassette and a loxP sequence were inserted between exons 13 and 14 and between exons 20 and 21, respectively. DTA, diphtheria toxin fragment A. B. Southern blot analyses using 5’ (left panel) and 3’ (right panel) external probes. XbaI (Left) and BamHI (Right) digested ES cell DNA were hybridized with probes that detect a 16.0 kb WT and 8.0 kb targeted alleles at the 5’ end (Left), or a 13.4 kb WT and a 6.2 kb targeted alleles at the 3’ end (Right). C. Confirmation of the Jmjd3 null allele by genomic PCR. PCR using primers spanning floxed region and Stra8-Cre allele are shown. Pgk1-Neo cassette was removed by Flp/Frt recombination because insertion of the cassette resulted in post-neonatal lethality similar to Jmjd3 knockout mice. D. Histology of control (F/-) and JMJD3 cKO (cKO) testis at 3-week of age. Scale bar: 50 µm. (TIFF) [file pone.0072689.s002.tiff]

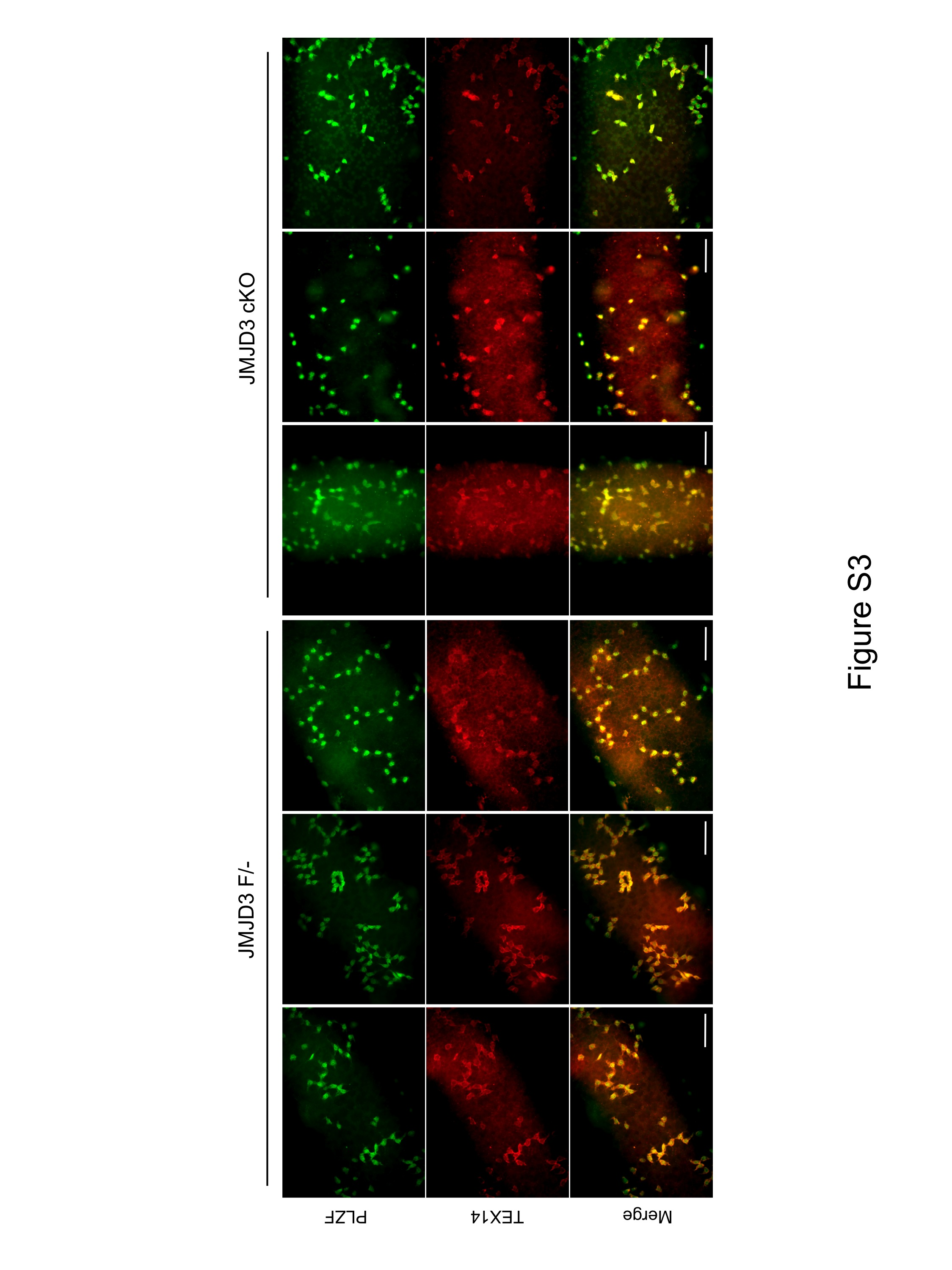

Supplement: Figure S3 — Visualization of spermatogonial chain with TEX14 and PLZF. Whole mount immuno-staining of seminiferous tubules for TEX14 and PLZF. Representative pictures of TEX14 and PLZF staining are shown. Scale bar: 50 µm. (TIFF) [file pone.0072689.s003.tiff]

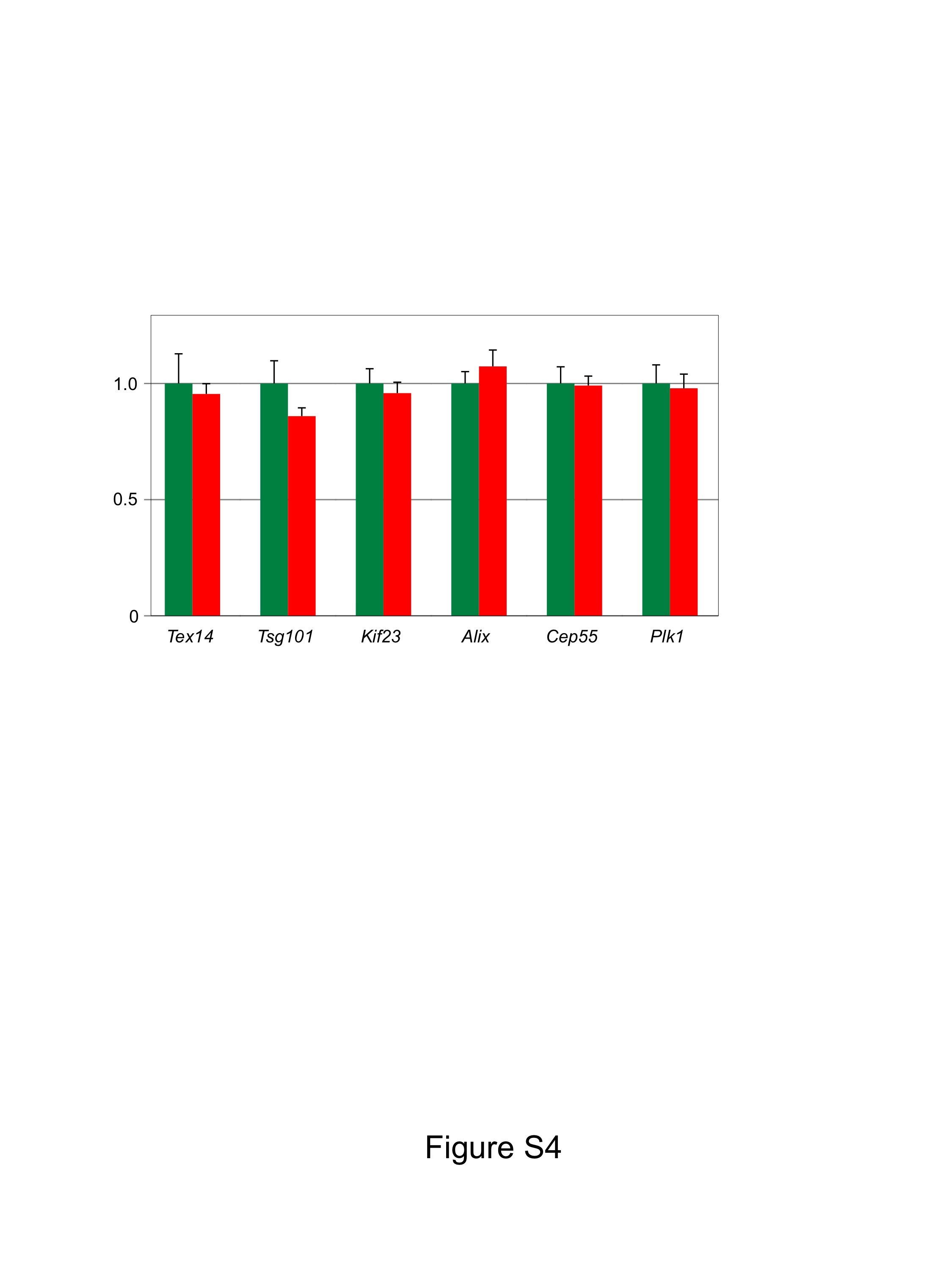

Supplement: Figure S4 — Effect of loss of JMJD3 for intercellular bridge formation. Relative expression of genes related for intercellular bridge formation (Tex14, Cep55, Alix, Tsg101, Alix, Kif23, Plk1) are shown. All expression levels were normalized to Gapdh expression. (TIFF) [file pone.0072689.s004.tiff]
