# Supplementary material for: H3K27 Demethylase, JMJD3, Regulates Fragmentation of Spermatogonial Cysts
Source: PLoS One. 2013 Aug 15;8(8):e72689. doi: 10.1371/journal.pone.0072689 (PMC3744460; doi:10.1371/journal.pone.0072689)
Supplement: Table S1 — Primer sequences used in the study. (PDF) [file pone.0072689.s005.pdf]

Table S1. Primer sequences used in the study

Gene name	Forward primer	Reverse primer
Plzf	CACACTCAAGAGCCACAAGC	ATCATGGCCGAGTAGTCTCG
Jmjd3	CTCTGGAACTTTCATGCCGG	CTTAGCCCCATAGTTCCGTTTG
Nanos2	AACTTCTGCAAGCACAATGG	CCGAGAAGTCATCACCAG
Ret	GGCTGTCCCGAGATGTTTATG	GACTCAATTGCCATCCACTTGA
Gfra1	TACCACCAGCATGTCCAATGAA	GTAGCTGTGCTTGGCTGGAACT
Stra8	ACAAGAGTGAGGCCCAGCAT	CCTCTGGATTTTCTGAGTTGCA
Neurog3	GCCTCATTGGAGGAATTCC	AGATGCTTGAGAGCCTCCAC
Taf4b	AGCCTAACAGCCACCAAACC	TGAATTCTCAGCGGCATG
Cdh1	ACCGATTCAAGAAGCTGGC	ACCATCCTAACACAGACAGTCC
Epcam	TGCTCCAAACTGGCGTCTAA	TCCCAGACTTGCTGTGAGTCA
Cd9	TGCATGCTGGGATTGTTCTTC	GGCGGCGGCTATCTCAA
Id4	GAGACTCACCCTGCTTTGCT	ATGCTGTCACCCTGCTTGTT
Eif1a	TAGCTCTCAGAAGCCAGGACTC	GCCTTTATTCTTTGGCATGATG
Ink4a	GTGTGCATGACGTGCGGG	GCAGTTCGAATCTGCACCGTAG
Arf	GCTCTGGCTTTCGTGAACATG	TCGAATCTGCACCGTAGTTGAG
Cdkn1a	GCAGATCCACAGCGATATCC	CAACTGCTCACTGTCCACGG
Cdkn1b	AAGGGCCAACAGAACAGAAG	GGATGTCCATTCAATGGAGTC
Clnd1	GTTCATTTCCAACCCACCC	CTCAGATGTCCACATCTCGC
Clnd2	GATCACCCACACTGATGTGG	ATGACGAACACGCCTCTCTC
Clnd3	ATGTCACAGCCATTCACCTG	CTGGTTGAGTGGGAAGGAAG
Clne	CTGGTTGAGTGGGAAGGAAG	TAGAGCACAGCATCTGCAGG
Tex14	AAATAGTAGGAGTATGGCGTCTG	CCATTTCAAGTGTGCCTCTC
Tsg101	ACATCCACGGTCAGAGTTGC	CCCTGTTGCTGTGTATGGTG
Alix	CGAGGAGCTCAGCAAACTGC	TGGGAACTTGGGTTCAATGG
Cep55	AAAGCAAATGGGGATCAAGG	TCCTTTCCCAGAGGTGATTTC
Kif23	TGACCAAGAATGCTGTGTGG	AGTTCCTTCTGGGTGGTGTG
Plk1	AGCAGCAGGAAACCTCTCAA	ACCACCGGTTCCTCTTTCTC
